# An unusual case of left renal artery compression: a rare type of median arcuate ligament syndrome

**DOI:** 10.1007/s00276-015-1478-8

**Published:** 2015-05-05

**Authors:** Agata Arazińska, Michał Polguj, Andrzej Wojciechowski, Łukasz Trębiński, Ludomir Stefańczyk

**Affiliations:** Department of Radiology, Medical University of Łódź, Kopcińskiego 22, 90-153 Lodz, Poland; Department of Angiology, Medical University of Łódź, Narutowicza 60, 90-136 Lodz, Poland

**Keywords:** Left renal artery compression, Median arcuate ligament syndrome, Hypertension, Computed tomography angiography

## Abstract

Compression from median arcuate ligament was observed during multidetector 64-row computed tomography in a Caucasian 30-year-old female. The patient was referred for examination to exclude anatomical pathologies causing hypertension. The examination demonstrated that left renal artery, which had its origin in the chest (at the level of upper one-third of Th12), was compressed as it passed by median arcuate ligament of the diaphragm. In addition, aortic compression and kinked shape was also revealed.

## Introduction

Median arcuate ligament syndrome (MALS), commonly called celiac artery compression syndrome (CACS), is a condition attributed to compression of celiac trunk (CT) and possibly celiac ganglia by median arcuate ligament (MAL). This pathology is accompanied by demonstrable lateral (poststenotic) dilatation of celiac trunk and resulting in a characteristic hook-shaped contour of the vessel [7]. It is a frequent finding in imaging studies performed for screening or diagnostic purposes. Most of the patients showing radiological features of this syndrome have no symptoms related to celiac trunk compression [5, 8]. A review of literature reveals that the prevalence of the syndrome is two per 100,000 patients and a female to male ratio is 2–3:1 [12]. It rarely causes ischemia or decreased blood flow to abdominal organs, leading to pain [4, 6]. Arterial constriction by the diaphragmatic crura has also been reported to involve multiple vessels [2, 9]. Symptoms of these pathologies are related to entrapped vessels.

We report the first case of coexistent compression of both left renal artery and aorta by median arcuate ligament with kinked aorta. This pathology was revealed in computed tomography angiography of the abdomen. The patient was referred for the examination due to hypertension.

## Case report

**Fig. 1:**
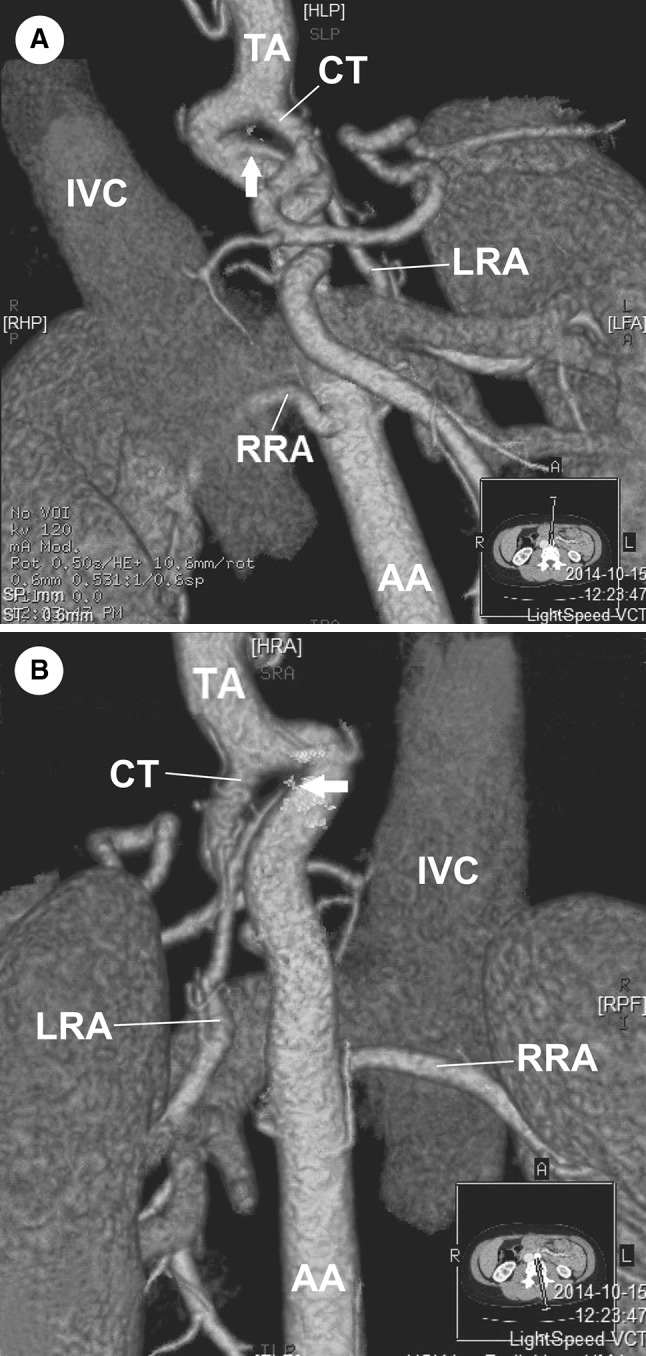
Abdominal computed tomography angiography, volume rendering. **a** Anterior view, **b** posterior view. *Arrow* origin of left renal artery, *AA* abdominal aorta, *CT* celiac trunk, *IVC* inferior vena cava, *LRA* left renal artery, *RRA* right renal artery, *TA* thoracic aorta

**Fig. 2:**
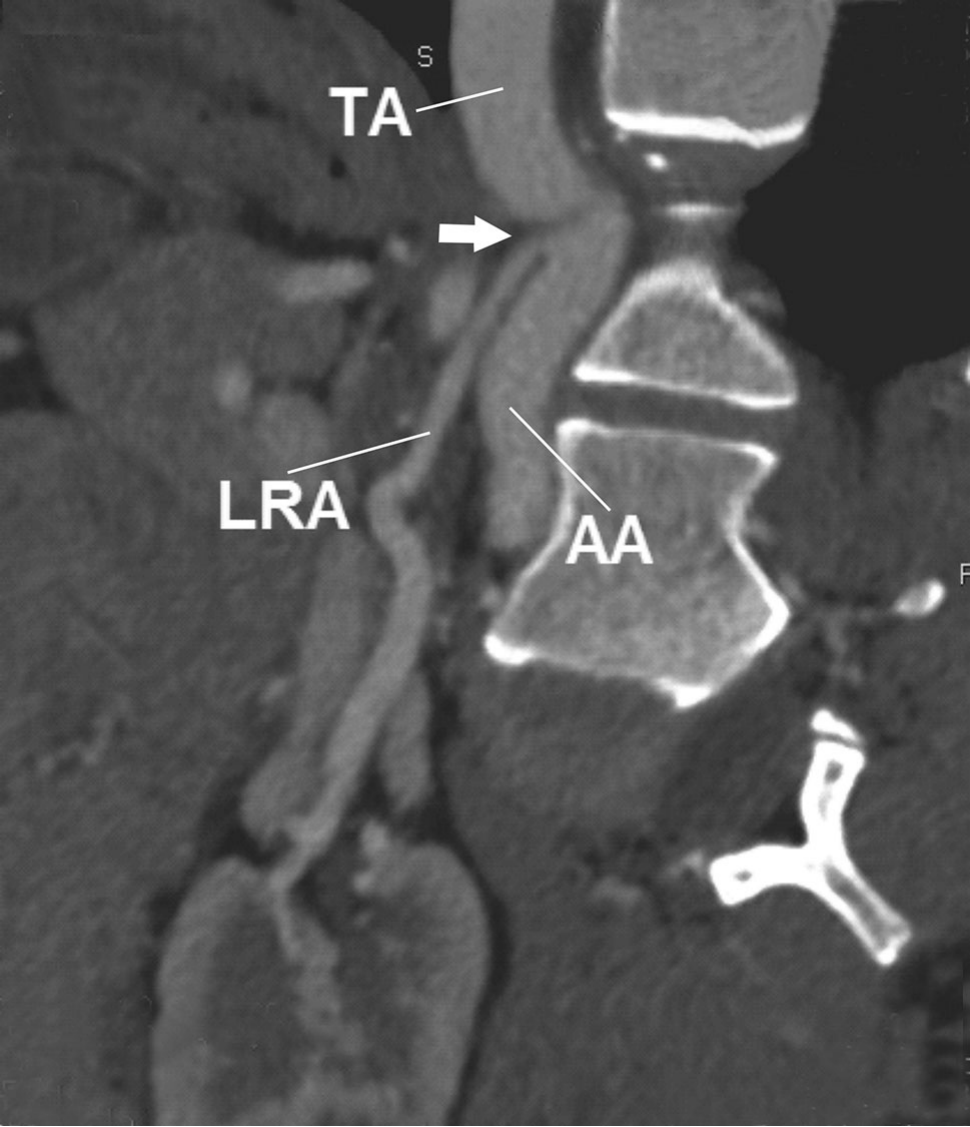
Abdominal computed tomography angiography, planar reconstruction. *Arrow* origin of left renal artery. *AA* abdominal aorta, *LRA* left renal artery, *TA* thoracic aorta

**Fig. 3:**
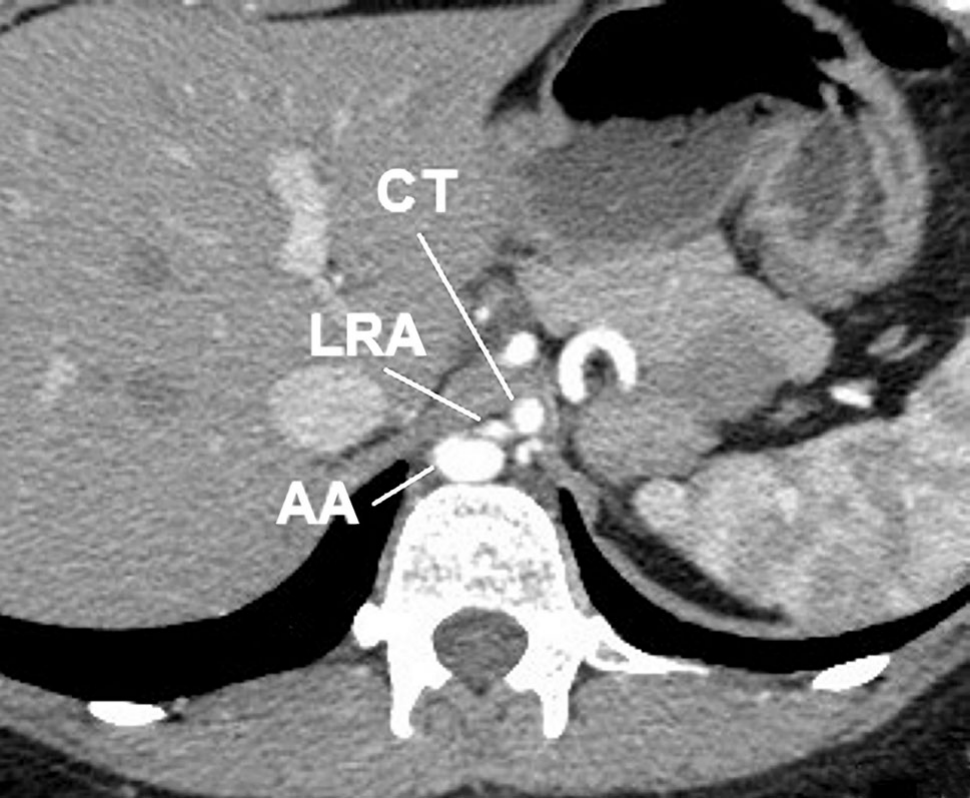
Abdominal computed tomography angiography, transverse scan. *AA* abdominal aorta, *CT* celiac trunk, *LRA* left renal artery

**Fig. 4:**
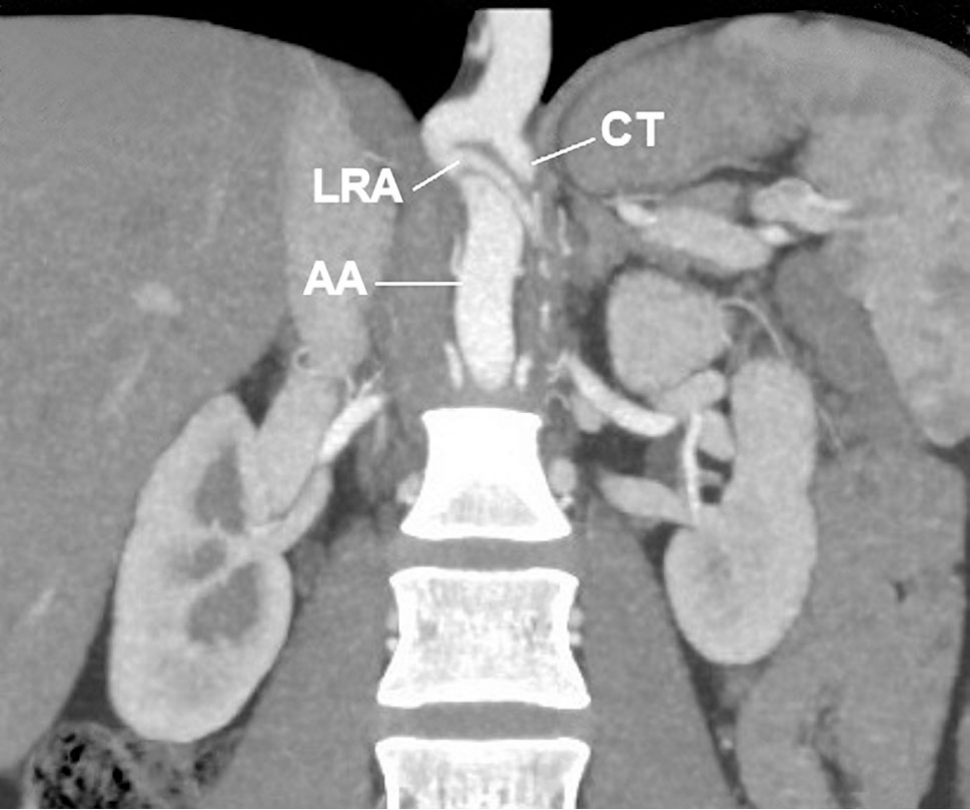
Abdominal computed tomography angiography, planar reconstruction. *AA* abdominal aorta, *CT* celiac trunk, *LRA* left renal artery

A 30-year-old Caucasian female was admitted to the nephrology out-patient clinic of our hospital due to hypertension. She was referred to radiology department for abdominal computed tomography angiography to exclude anatomical pathology which may have resulted in hypertension. Computer tomography angiography (CT-64-row MDCT scanner, LightSpeed VCT, GE, Waukesha, Wisconsin, US) revealed numerous arterial anomalies. Thoracic aorta was kinked and compressed by the MAL (Figs. 1, 2, 3, 4). It measured 8 mm in diameter at the point of narrowing, which equals to 40 % of its lumen. From the kinked part of the aorta, left renal artery (LRA) had its origin in the thorax, at the level of upper one-third of Th12 (Figs. 2, 3, 4). This vessel also was constricted in its origin by MAL (Figs. 2, 3). In abdomen, the left renal artery had segmentally arcuate and tortuous shape (Figs. 1, 2). The LRA lumen decreased to 2 mm in diameter at the point of narrowing which equals to 60 % (Figs. 2, 3). In addition, CT arose also in the thorax at the level of upper one-third Th12, but it was not affected by stenosis (Figs. 3, 4). Due to thoracic aorta kinking origins of CT and LRA were 10 mm away from each other, which lead to 3 mm distance perpendicularly. Stenosis of the abdominal aorta was not detected and the vessel measured 13.5 mm in diameter at the level of right renal artery’s (RRA) origin. Comparatively, the lumen of the RRA measured 4.5 mm in diameter and was free of any abnormality. Level of origin was typical (L2). The distance between origins of LRA and RRA amounted to 62 mm. There were no thoracolumbar vertebral anomalies. No surgical procedure has been performed due to patient’s disapproval. For such a reason, pharmacological treatment was introduced.

## Discussion

The MAL is a tendinous band that connects the medial borders of the diaphragm crura on either side of the aortic hiatus. It is commonly located posterior and superior to the origin of the CT. The ligament is highly variable, with its appearance ranging from a well-defined ligamentous mass to an amorphous area of connective tissue [5]. Usually, the MAL crosses the aorta at the level of L1; therefore, it is located above the origin of the CT. However, in 10 to 24 % of the overall population, inadequate caudal migration of the artery during embryogenesis or low insertion of the ligament produces compression of the proximal part of the CT [4, 7].

The diaphragmatic crura have also been reported to cause extrinsic compression of the aorta, mesenteric arteries, lumbar arteries and both renal arteries [2, 5, 8, 9]. The celiac trunk compression is much more common than others; however, more than one artery can be involved in some patients [9]. There have been isolated cases reported in which the MAL compressed both the CT and superior mesenteric artery (SMA) [9]. Our patient’s examination demonstrated stenosis of the vessels caused by the MAL. The thoracic aorta as well as the LRA was compressed by the MAL, but diameter of lumen of the CT was unchanged. Secondary to compression, aorta had kinked shape. This configuration of vessels and stenosis configuration makes our patient’s case exceptional.

Anatomic variations of the celiac trunk are frequent and may be related to its origin and its collaterals. Usually, CT originates from abdominal aorta at the level of the upper one-third of the first lumbar vertebra. However, studies reveled that CT was found to originate between the upper one-third of Th11 and middle one-third of L2 [10–13]. In our patient, CT arose at the level of upper one-third Th12, but it was not affected by stenosis. Several cases of unusual origin of the celiac trunk in the thorax were reported and also did not reveal compression of CT nor stenosis at the level of aortic hiatus and MAL [10–12].

Coexistence of a coarctation of thoracic aorta with supradiaphragmatic origin of the celiacomesenteric trunk (CMT) was presented by Lee et al. [11]. The descending aorta at the level of Th11 demonstrated severe focal stenosis, but neither thickening nor enhancement of their walls was found. The CMT originated just above the stenosis. Authors presumed that the atypical high origin of the CMT is related to the stenosis of aorta in fetal development. In Lee et al.’s [11] opinion, an isolated vascular abnormality of a descending aorta may be congenital or acquired in course of inflammatory disease such as Takayasu arteritis [11].

Renal artery entrapment by the diaphragmatic crus was first described by D’Abreu [3] who reported two cases proven by surgery in 1962. Since its first description, only a few cases have been reported in the literature [1, 9, 15, 16, 19]. However, none was accompanied by stenosis and kinked aorta. Such configuration may secondarily predispose to development of atherosclerosis at the wall of the surrounding arteries. Thony et al. [17] have reported that renal artery entrapment should be suspected each time angiography shows a renal artery parallel to the aorta in the proximal part of its course. Our findings confirmed this observation. Usually, renal artery compression is caused by fibres forming part of the crus of the diaphragm or the psoas muscle impinging on the renal artery by verticalisation of the root of the renal artery [16, 19]. This results in its stenosis and arcuate or tortuous course. This anomaly is also associated with a high origin of the renal artery from the aorta and is more common on the left side. The mechanism evoked is an anomaly of the kidney migration [16].

In cases of renal artery compression by the MAL, patients usually suffer from hypertension [5]. Renal artery stenosis accounts for about 1 % of patients with hypertension, but its incidence rises to 30 % in cases of refractory hypertension. The two major causes are atherosclerosis and fibromuscular dysplasia. Extrinsic compression of the renal artery by the diaphragmatic crura is a very rare cause of hypertension [5].

The diagnosis of median arcuate ligament syndrome can be established with several different imaging modalities such as conventional angiography, CT angiography (CTA), MR angiography (MRA), Doppler US and intravascular ultrasound (IVUS) [9, 14]. Since MALS was first described, a number of different treatment options have been explored. Currently, the most popular option of treatment is by laparoscopic division of the MAL [18].

Understanding the topography and variations of the abdominal vessels is important for diagnosis and treatment. To the best of our knowledge, the left renal artery and aortic entrapment caused by compression created by median arcuate ligament coexisting with kinking aorta has not been previously reported. It makes this case particularly important especially in surgery and diagnosis of hypertension.
